# Plant species influences the composition of root system microbiome and its antibiotic resistance profile in a constructed wetland receiving primary treated wastewater

**DOI:** 10.3389/fmicb.2024.1436122

**Published:** 2024-07-24

**Authors:** Valentina Riva, Lorenzo Vergani, Ahmed Ali Rashed, Aiman El Saadi, Raffaella Sabatino, Andrea Di Cesare, Elena Crotti, Francesca Mapelli, Sara Borin

**Affiliations:** ^1^Department of Food, Environmental, and Nutritional Sciences (DeFENS), University of Milan, Milan, Italy; ^2^National Water Management and Irrigation Systems Research Institute, National Water Research Center, Shoubra meuip El-Kheima, Egypt; ^3^National Research Council of Italy – Water Research Institute (CNR-IRSA) Molecular Ecology Group (MEG), Verbania, Italy; ^4^National Biodiversity Future Center (NBFC), Palermo, Italy

**Keywords:** phytodepuration, plant-microbe interactions, water reuse, *Phragmites australis*, *Typha domingensis*, antibiotic resistance genes

## Abstract

**Introduction:**

Constructed wetlands (CWs) are nature-based solutions for wastewater treatment where the root system microbiome plays a key role in terms of nutrient and pollutant removal. Nonetheless, little is known on plant-microbe interactions and bacterial population selection in CWs, which are mostly characterized in terms of engineering aspects.

**Methods:**

Here, cultivation-independent and cultivation-based analyses were applied to study the bacterial communities associated to the root systems of *Phragmites australis* and *Typha domingensis* co-occurring in the same cell of a CW receiving primary treated wastewaters.

**Results and discussion:**

Two endophytic bacteria collections (*n* = 156) were established aiming to find novel strains for microbial-assisted phytodepuration, however basing on their taxonomy the possible use of these strains was limited by their low degrading potential and/or for risks related to the *One-Health* concept. A sharp differentiation arose between the *P. australis* and *T. domingensis* collections, mainly represented by lactic acid bacteria (98%) and Enterobacteriaceae (69%), respectively. Hence, 16S rRNA amplicon sequencing was used to disentangle the microbiome composition in the root system fractions collected at increasing distance from the root surface. Both the fraction type and the plant species were recognized as drivers of the bacterial community structure. Moreover, differential abundance analysis revealed that, in all fractions, several bacteria families were significantly and differentially enriched in *P. australis* or in *T. domingensis*. CWs have been also reported as interesting options for the removal of emerging contaminants (e.g, antibiotic resistance genes, ARGs). In this study, ARGs were mostly present in the rhizosphere of both plant species, compared to the other analyzed fractions. Notably, qPCR data showed that ARGs (i.e., *erm*B, *bla*_TEM_, *tet*A) and *intl*1 gene (integrase gene of the class 1 integrons) were significantly higher in *Phragmites* than *Typha* rhizospheres, suggesting that macrophyte species growing in CWs can display a different ability to remove ARGs from wastewater. Overall, the results suggest the importance to consider the plant-microbiome interactions, besides engineering aspects, to select the most suitable species when designing phytodepuration systems.

## 1 Introduction

Constructed wetlands (CWs) are engineering ecosystems worldwide used for water depuration purposes. The advantages of these systems include (i) the possibility to treat different kinds of wastewater in a cost-efficient way, (ii) the limited operation and maintenance requirements and (iii) the environmental quality and landscape conservation (Parde et al., 2021). CWs have been adopted for decades (Vymazal, 2022) and have been contextualized within the Water-Energy-Food nexus (FAO, 2014) as robust nature-based solution (Carvalho et al., 2022). CWs can be applied in wastewater treatment either as primary treatment or as secondary/tertiary treatment process. As primary treatment, CWs are mostly used in low-income countries (Zhang et al., 2014) but they are also a valid solution for small communities located in industrialized countries, such as those living in remote mountainous areas (Stentella et al., 2023) and in rural arid regions (Troiano et al., 2018; Stefanakis, 2022). Downstream the primary treatment, they can be exploited for the removal of recalcitrant pollutants present in industrial wastewaters or emerging organic contaminants (EOCs, like antibiotics and other pharmaceuticals) present in urban wastewaters (Carvalho, 2021). In fact, while conventional wastewater treatment plants are inadequate for removing these contaminants from wastewaters (Gorito et al., 2017), many studies demonstrated the efficiency of CWs, as reviewed by Yan et al. (2022). CWs are complex ecosystems which functioning relies on both physicochemical and biological processes including photodegradation, sedimentation, volatilization, sorption and biological degradation (Kadlec and Wallace, 2009). The plant root system, in particular, provides in CWs an ecological niche for the development of microbial communities that are pivotal for nutrient and pollutant removal from wastewaters and can also sustain plant growth (Faulwetter et al., 2009). Moreover, different studies showed that the administration of specific bacteria to the plants used in CWs represents a promising strategy to boost phytodepuration performances (Syranidou et al., 2016; Prum et al., 2018; Rehman et al., 2018; Riva et al., 2019), making microbial assisted phytodepuration an interesting option.

In this context, a deeper knowledge of plant-associated microbiomes in CWs would facilitate the implementation of tailored isolation trials aiming at culturing the most interesting bacteria for biotechnological application in phytodepuration. In the last decade, a plethora of scientific articles reported data on CWs microbial community composition, providing a general snapshot of the main bacterial phyla (i.e., Proteobacteria, Bacteroidetes, Actinobacteria and Firmicutes) involved in phytodepuration processes (Wang et al., 2022). However, the ability of different plant species to recruit specific bacterial communities when receiving the same wastewater and growing under the same conditions is a neglected aspect for the choice of the plant species to use in a CW, currently related exclusively to engineering aspects.

To improve the knowledge about the tight interactions occurring in the plant holobiont involved in phytodepuration processes, the present work studied the bacterial communities associated to *Phragmites australis* and *Typha domingensis* plants co-occurring in an Egyptian CW receiving primary treated wastewater of mixed origins (i.e., agricultural, municipal and industrial), implementing both cultivation-based and cultivation-independent analyses. The occurrence of antibiotic resistance genes (ARGs) and the class 1 integrons (targeting the integrase gene *intl*1, known to have a strong positive correlation with ARGs, Fang et al., 2017) in the plant-associated bacterial communities was also investigated. In fact, antibiotic resistance diffusion through irrigation is a possible criticism of wastewater treatment technologies considering the new European legislation about water reuse (2020/741) which now considers antimicrobial resistance determinants among other emerging contaminants. A recent review article showed the efficacy of CWs in removing ARGs and antibiotic resistant bacteria from wastewaters (Hazra et al., 2022), however, the rhizosphere has been indicated as a hot spot for Horizontal Gene Transfer (HGT) and the microbiota associated to CW plants as a possible ARG reservoir, due to the selective pressure exerted by antibiotics (Riva et al., 2020). For this reason, a further objective of the present work was the investigation of the possible differences occurring among *Phragmites australis* and *Typha domingensis* associated microbiota in terms of antibiotic resistance profile.

## 2 Materials and methods

### 2.1 Site description and sampling

*Phragmites australis* and *Typha domingensis* root systems were collected from the Manzala Lake Engineered Wetland, a large pilot scale Free Water Surface Constructed Wetland (FWS-CW) located in Egypt. The system was constructed in 2000 to improve the water quality of Bahr El Baqar drain, which is located at the northeast edge of the Nile Delta. It consists of three lifting pumps from the drain, two sedimentation basins as primary water treatment, and ten FWS-CWs beds as secondary water treatment. *Phragmites australis* and *Typha domingensis* grow combined in FWS-CWs at high-density (the schematic layout of the system is shown in Supplementary Figure S1A). Each FWS-CW cell has a dimension of 250 × 50 × 0.5 meters and a retention time of 2 days. The CW receives primary treated waters of multiple types (mainly agricultural drainage water, mixed with wastewaters produced by small factories producing milk and dairy products, municipal and industrial wastewaters). The water quality improvement gained with this system was monitored from 2004 to 2016, demonstrating successful results in reducing nutrient, fecal coliform and heavy metal loads. The water quality parameters of the influent and the effluent measured in 2016 are shown in Supplementary Table S1. The measurements of emerging contaminant concentrations, such as pharmaceuticals and antibiotic resistance determinants, was not included in the characterization since their removal was out of the primary scope of this CW system.

**Figure 1:**
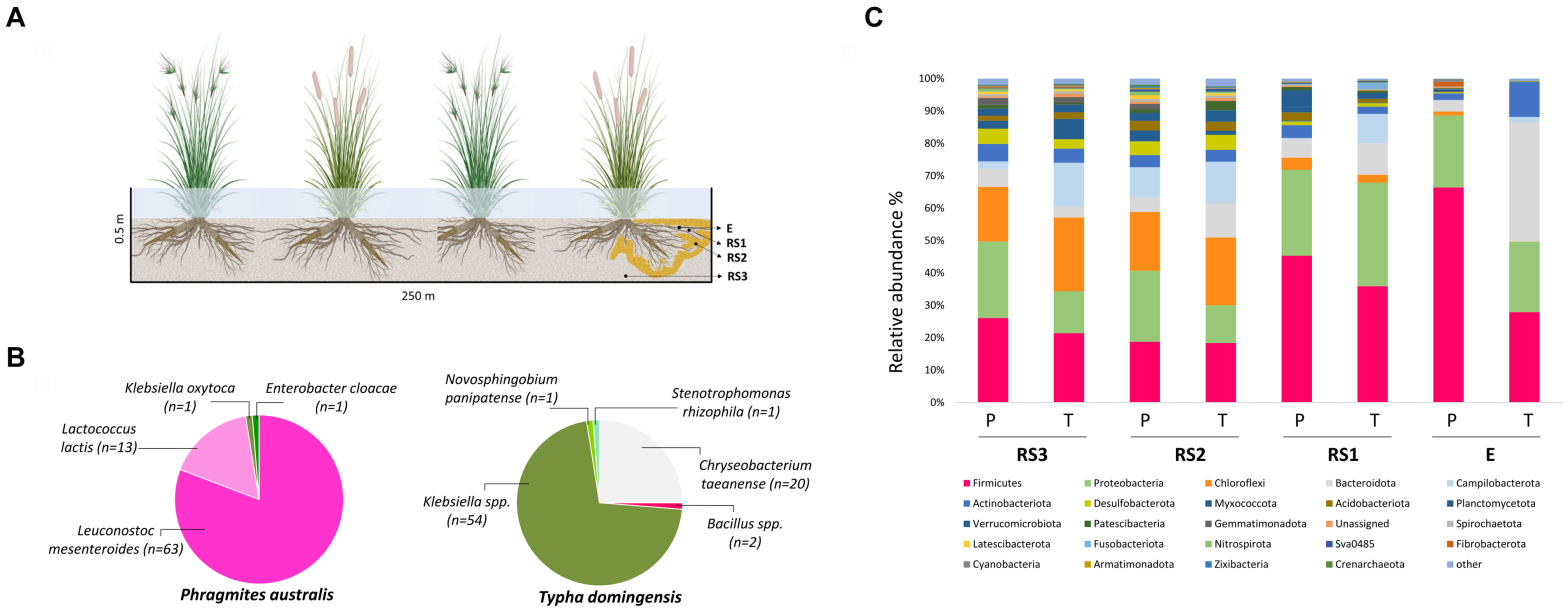
**(A)** Schematic diagram of sample collection. E = endosphere; RS1 = Rhizosphere (Root System 1); RS2 = Root System 2; RS3 = Root System 3; **(B)** Taxonomic distribution of 16S rRNA gene sequences of culturable root-endophytic bacteria isolated from *Phragmites australis* and *Typha domingensis* plants. **(C)** Bacterial microbiota composition of *Phragmites australis* (P) and *Typha domingensis* (T) across fractions as detected by 16S rRNA amplicon sequencing analysis. Relative abundance of the main phyla is reported as mean of replicates (*n* = 3) for each category (fraction per plant species). Taxonomic groups with relative abundance < 1% over the total dataset are summed and reported as “other.”

In December 2016, root system samples were collected from three different plants of *P. australis* and *T. domingensis* growing at the entrance of the same FWS-CW cell. Temperature variation in the area where the FWS-CW is located is reported in Supplementary Figure S1B. Root (E) and root system (RS) samples were collected using sterile tools, stored at 4°C and shipped to the laboratories of the University of Milan within 48 h. Codes RS1-RS2-RS3 correspond to the root system fractions sampled at increasing distance from the root surface according to the scheme represented in the Figure 1A. RS1 is the soil tightly attached to the roots (i.e., rhizosphere), RS2 indicates the soil sampled close to the roots, while RS3 is the portion of the root system soil sampled at 50 cm depth of the FWS-CW cell more loosely influenced by the roots.

### 2.2 Endophytic bacteria isolation

To obtain the endophytic bacterial collections from *P. australis* and *T. domingensis* samples, roots collected from 3 plant specimens (around 1 g/specimen) were pooled, vigorously washed in physiological solution (0.9% NaCl) for 10 min and surface-sterilized in 70% ethanol for 3 min, 1% sodium hypochlorite for 5 min, 70% ethanol for 30 s and rinsed four times for 2 min in sterile distilled water before a final washing step in sterile distilled water for 30 min. A 100 μL of the last rinsing water was plated on the same medium later used for bacteria isolation (medium 869 diluted 1:10869; Barac et al., 2004), to confirm the effectiveness of root surface sterilization. Roots were then smashed with sterile pestle and mortar. One gram of the resulting root tissue homogenate was suspended in 9 mL of physiological solution, serially diluted and plated in triplicate on 1:10869 medium supplemented with cycloheximide 0.1 g/L to prevent fungal growth. After incubation at 30°C for 24 h, bacterial colonies were randomly picked and spread three times on the same medium to obtain pure cultures. In total, 156 root endophytic bacteria were isolated from *T. domingensis* (*n* = 78) and *P. australis* (*n* = 78), and cryopreserved in 25% glycerol stocks at −80°C. The isolates were labeled with codes including the sampling site (‘CWE’ for Constructed Wetland in Egypt), the plant species (‘P’ for *P. australis* and ‘T’ for *T. domingensis*), the medium used for the isolation (‘8’ for medium 869 diluted 1:10) and the fraction (‘E’ for endosphere) followed by progressive numbers.

### 2.3 Bacterial taxonomic identification and genotyping

The genomic DNA of the bacteria isolated from *P. australis* and *T. domingensis* root endosphere was extracted through a phenol:chloroform protocol (Sambrook and Russell, 2006) and the bacterial isolates were identified through 16S rRNA gene amplification and partial sequencing (Macrogen, Rep. of South Korea) as previously described (Mapelli et al., 2013). The nucleotide sequences were edited in Chromas Lite 2.01 and compared to those deposited in the NCBI database (https://blast.ncbi.nlm.nih.gov/Blast.cgi?PAGE_TYPE=BlastSearch). 16S rRNA gene partial sequences were deposited in the NCBI GenBank database under accession numbers OQ942931 - OQ943084.

The bacterial collections were then de-replicated by 16S-23S rRNA Intergenic Transcribed Spacer PCR (ITS-PCR) and BOX-PCR and combining the results of both fingerprinting analyses as previously described (Chouaia et al., 2010).

### 2.4 Metagenomic DNA extraction

Metagenomic DNA was extracted from root endosphere (E; *n* = 3 for each plant sp.) and the three different root system fractions (RS1-RS2-RS3; *n* = 3 for each fraction and each plant sp.) collected at increasing distance from root surface. For both plant species, the RS1 fraction was obtained by thoroughly washing root samples with 20 mL of sterile physiological solution (RS1 tube). After vortexing for 20 min at high speed, root samples were put in a new 50 mL tubes with 20 mL of sterile physiological solution and shaken again. The washing solution was added to the RS1 tube and after centrifugation at 5000 rpm at 4°C for 10 min the liquid was discharged. Root samples were vigorously washed four times with sterile distilled water for 3 min and surface-sterilized with 70% ethanol for 5 min, 1% sodium hypochlorite for 5 min, 70% ethanol for 3 min and rinsed 5 times for 3 min in sterile distilled water. Surface sterilized root samples (E) were then crushed using liquid nitrogen and 100 mg of samples material were homogenized using the TissueLyser II (Qiagen). The metagenomic DNA was extracted with Dneasy Plant Mini Kit (Qiagen) from the endosphere fraction (E) or using the Dneasy PowerSoil Kit (Qiagen) for all RS fractions.

The DNA concentration of each sample was assessed using a Qubit™ fluorometer with dsDNA HS kit (Thermo Fisher Scientific Inc.).

### 2.5 Antibiotic resistance characterization

#### 2.5.1 Minimal inhibitory concentration of endophytic bacteria isolates

Selected isolates belonging to the group of lactic acid bacteria and to the genera that include clinically relevant species were characterized for their resistance to different antibiotics chosen from a panel that includes ampicillin, vancomycin, gentamycin, kanamycin, streptomycin, erythromycin, clindamycin, tetracycline, chloramphenicol and ciprofloxacin (Supplementary Table S2). The tested antibiotics were selected according to EFSA guidelines (2012) for lactic acid bacteria, and CLSI guidelines (2014) for the rest of the bacteria collection (*Enterobacteriaceae* and other non-*Enterobacteriaceae*). According to the results of fingerprinting analyses, one representative strain per each ITS-BOX cluster was selected for the test: 4 *Leuconostoc mesenteroides*, 6 *Lactococcus lactis*, 11 *Klebsiella* spp., 1 *Enterobacter cloacae* and 10 *Chryseobacterium taeanense* strains were tested. The Minimal Inhibitory Concentration (MIC) was determined by micro-dilution methods (Andrews, 2001; EUCAST, 2003), using standardized LAB susceptibility test medium (LSM) broth and a mixture of IsoSensitest broth medium (Thermo Scientific) (90%) and MRS broth medium (Sigma-Aldrich) added of 1 mL/L Tween80 (Sigma-Aldrich) (10%) (Arioli et al., 2013; Noda et al., 2019) for *Leuconostoc* and *Lactococcus* strains, respectively. CAMHB (Cation Adjusted Mueller Hinton Broth) and MHB medium were used for the MIC tests on *Enterobacteriaceae* and *Chryseobacterium* strains, respectively (CLSI, 2014; EUCAST, 2021). Each strain was incubated, in triplicate, in absence (control) and in presence of each antibiotic at seven different concentrations (see Supplementary Table S2 for concentration ranges). The test was performed in 96-well plates with a final volume of 200 μL and starting with a bacterial concentration of 5 × 10^5^ cell/ml. Plates were incubated at 30°C and after 24 h the optical density (OD) was measured at 610 nm using a spectrophotometer (Tecan Infinite F200Pro). MIC results were interpreted according to EFSA and CLSI-approved cut-off values.

#### 2.5.2 Amplification and quantification of antibiotic resistance genes (ARGs) and *intl*1 gene in *Typha* and *Phragmites* root systems

Metagenomic DNA extracted from *Typha* and *Phragmites* root systems was used as template for qualitative PCRs targeting different ARGs and the *intl*1 gene. The presence of genes encoding for the resistance to tetracycline (*tet*A), β-lactams (*bla*_TEM_, *bla*_CTXM_, *bla*_OXA_), aminoglycosides (*str*B), fluoroquinolones (*qnr*S), sulfonamides (*sul*II), macrolide-lincosamide-streptogramin (MLS) (*erm*B) and colistin (*mcr-*1) was assessed by PCRs according to the thermal protocol indicated by Di Cesare et al. (2015): 95°C for 3 min, 30 cycles of 95°C for 30 s, annealing temperature (AT) specific for each gene and primer pair for 1 min, 72°C for 30 s and a final extension of 72°C for 7 min. Details about the primer pairs used in this study and the respective AT and amplicon sizes are summarized by Fonti et al. (2021). For both plant species, DNA extracted from RS1 fraction was also used as template for quantitative PCR (qPCR) targeting 16S rRNA gene, ARGs and the *intl*1 gene, executed in polypropylene 96-well plates on a BIORAD CFX Connect™ Real-Time PCR Detection System. ARGs and the *intl*1 gene were quantified with the conditions described by Di Cesare et al. (2015) and Di Cesare et al. (2016). 16S rRNA gene was quantified according to Vergani et al. (2024). Each qPCR assay included triplicate reactions per DNA sample and the appropriate set of standards and for all assays qPCR parameters are specified in Supplementary Table S3. qPCR products were tested for the specificity of the amplification by melting profile analysis. qPCR data were expressed as copy numbers/g of soil. T-student test was performed to compare qPCR results obtained on the DNA extracted from *Phragmites* and *Typha* samples.

### 2.6 16S rRNA gene amplicon sequencing and metataxonomic analysis

16S rRNA gene amplicon sequencing was obtained at Macrogen Korea by applying Illumina MiSeq sequencing of the V3-V4 hypervariable regions (Klindworth et al., 2013) of the bacterial 16S rRNA gene using the metagenome extracted from E and RS fractions as template. Peptide nucleic acid (PNA) clamps (Eurogentec, 0.75 μM per reaction) were used to minimize the amplification of DNA from the plant’s mitochondria and plastids from the endosphere fractions (Lundberg et al., 2012).

The 16S rRNA gene amplicon sequence data were processed using QIIME2 version 2022.2 (Bolyen et al., 2019) software. The DADA2 workflow was followed to assemble the reads and to perform denoising, dereplication and chimera-filtering following the default settings (Callahan et al., 2016). Forward and reverse reads were truncated to the length of 250 and 230 bp, respectively, and trimmed to remove primers and adapters. Taxonomy was assigned by using Silva v138 (Quast et al., 2013) to create a database of Amplicon Sequence Variants (ASVs) (Callahan et al., 2016). Adequacy of sequencing depth was evaluated performing rarefaction analysis on the alpha diversity observed on the obtained ASVs using QIIME2. The sequence reads were deposited in the NCBI SRA database under the BioProject ID PRJNA1055434.

Prior to further analysis, the ASVs relative to plastids and mitochondrial were deleted from the dataset, as well as the ASVs representing less than 0.005% of the dataset. Alpha diversity indices were calculated on the ASV table using the PAST software (Hammer et al., 2009). For the comparison of alpha diversity data between the different fractions, ANOVA and Tukey–Kramer tests were applied in the R software 4.2.0 (R Core Team, 2022), while for comparing data of the same fraction between the two plant species, t-student test was performed. A principal coordinate analysis (PCoA) was used to assess the phylogenetic β-diversity based on Bray–Curtis distance matrix on the normalized (log transformed) ASV table. Significant differences in bacterial community composition according to the factors ‘plant species’, ‘fraction’ and their interactions were investigated by PERMANOVA. Statistical analyses were conducted in PRIMER v. 6.1, PERMANOVA+ for PRIMER routine (Anderson et al., 2008). To identify the 16S rRNA gene sequences (classified at family level) which distribution was significantly influenced by the plant species, a differential abundance analysis was performed using quasi-likelihood F-test and the sequencing depth was standardized using the relative log expression (RLE) method as implemented in the R package EdgeR version 3.11 (Robinson et al., 2009; Wörner et al., 2016). The Tax4Fun function of MicrobiomeAnalyst 2.0 (Lu et al., 2023) was used to infer metabolic properties in the genera retrieved in the samples based on the Kyoto Encyclopedia of Genes and Genomes (KEGG) database (Aßhauer et al., 2015): the data were analyzed applying ANOVA and Tukey–Kramer test in the R software 4.2.0 (R Core Team, 2022).

## 3 Results

### 3.1 Characterization of the culturable endophytic bacteria associated to *Phragmites australis* and *Typha domingensis*

The culturable endophytic bacteria isolated from *T. domingensis* and *P. australis* (*n* = 156) were taxonomically identified aiming to obtain strains of potential interest for biotechnological application in phytodepuration systems. As showed in Figure 1B, the two plant species selected very different assemblages of cultivable bacterial populations and both collections were characterized by a low phylogenetic diversity. The collection retrieved from *T. domingensis* endosphere was mainly composed by *Klebsiella* spp. (69.2% of the collection) and *Chryseobacterium taeanense* (25.6%), while *Bacillus* spp. (2.6%) and *Novosphingobium panipatense* and *Stenotrophomonas rhizophila* (1.3% each) represented the minority of the isolates (Figure 1B). The *P. australis* collection included the bacterial species *Leuconostoc mesenteroides* (81%), *Lactococcus lactis* (17%), *Klebsiella oxytoca* and *Enterobacter cloacae*, the latter accounting each for 1% of the total collection (Figure 1B). All isolates were genotypically characterized combining the results of two fingerprinting analyses, which allowed to identify 12 and 23 ITS-BOX profiles for the *P. australis* and *T. domingensis* collections, respectively (Supplementary Table S4). Aiming to investigate the antibiotic resistance profile of the culturable endophytes associated to the two plant species, one representative strain per each genotype (*n* = 32) detected in the most abundant cultured genera (i.e., *Klebsiella*, *Chryseobacterium*, *Leuconostoc* and *Lactococcus*) has been selected for MIC tests on different antibiotics. The results showed that lactic acid bacteria strains were resistant to two out of nine antibiotics tested (Supplementary Table S5). All tested *L. mesenteroides* strains were resistant to ampicillin, while *L. lactis* strains were resistant to ampicillin (67% of the tested isolates) and streptomycin (100%). Conversely, the MIC assays indicated that all lactic acid bacteria were susceptible to vancomycin, gentamycin, kanamycin, erythromycin, clindamycin, tetracycline and chloramphenicol (Supplementary Table S5A). All *Klebsiella* spp. strains showed resistance toward ampicillin and were susceptible to gentamycin, kanamycin, tetracycline, streptomycin and chloramphenicol (Supplementary Table S5B). On the contrary, *Chryseobacterium taeanense* isolates showed a wide resistance profile, being resistant to all the five tested antibiotics (ampicillin, gentamycin, chloramphenicol, clindamycin and vancomycin) (Supplementary Table S5C).

### 3.2 Phylogenetic composition and diversity of bacterial communities associated to *Phragmites australis* and *Typha domingensis*

Bacterial community composition and diversity in the root systems of *P. australis* and *T. domingensis* were studied by a cultivation-independent approach, using 16S rRNA gene amplicon sequencing. Rarefaction curves were calculated showing that the sequencing effort was sufficient to capture the most abundant and rare taxa in each sample (Supplementary Figure S2). A total of 2,015,504 high-quality sequences classified in 950 unique ASVs were obtained in the 24 analyzed samples (Supplementary Table S6A). Alpha-diversity analysis on the bacterial communities indicated that the richness (i.e., number of ASVs) and the Shannon diversity indices increased moving from the endosphere to the RS3 fractions in both the plant species and, coherently, the dominance index decreased (Supplementary Figure S3). For endosphere (E) and rhizosphere (RS1) fractions, these results were supported by statistical analysis (Tukey–Kramer test, *p* values <0.05) while the changes were less sharp looking at the samples of the RS2 and RS3 fractions. Additionally, alpha diversity indices related to E fraction resulted significantly different between the two plant species (t-student test, p values <0.05): *T. domingensis* showed significantly lower richness and Shannon diversity indices values than *P. australis*, and a significantly higher dominance value.

**Figure 2:**
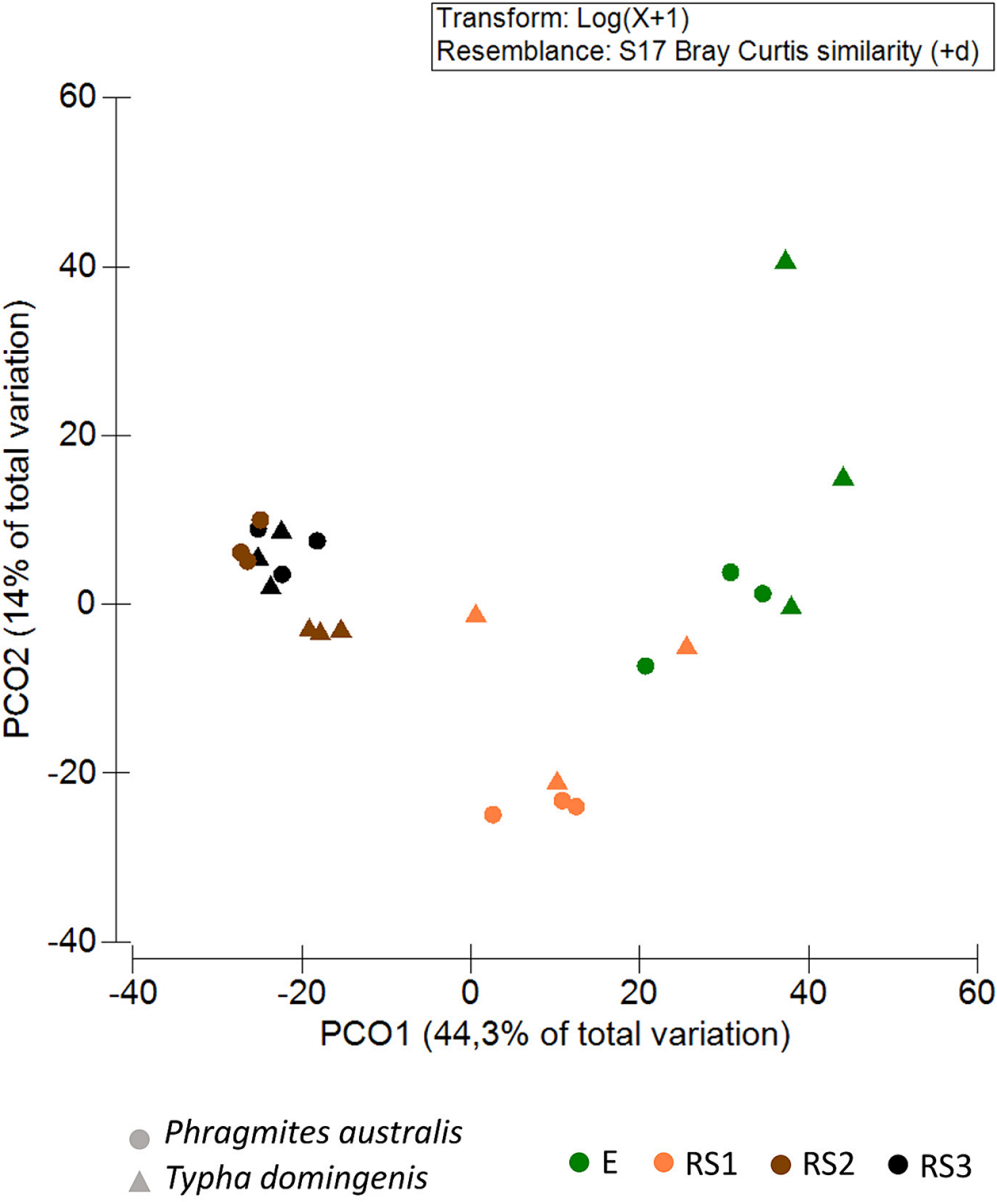
Bacterial community beta-diversity patterns. Principal coordinates analysis (PCoA) of the bacterial communities inhabiting the *Phragmites australis* and *Typha domingensis* root endosphere (E) and root system (RS1, RS2, RS3) fractions. PCoA was calculated from the ASV table generated by 16S rRNA amplicon sequencing. Colors indicate plant fractions, and shapes indicate plant species.

Principal Coordinates Analysis (PCoA) showed the clustering of bacterial communities along Axis 1 (44.3% of total variance) according to the fraction types. For both the plant species, the PCoA revealed a spatial niche separation (Figure 2) between the bacterial communities tightly connected to the plant roots (E and RS1) and those more loosely attached to the core of the root systems (RS2, RS3), as confirmed by the PERMANOVA pair-wise test (Supplementary Table S7A). Overall, the composition of the bacterial communities colonizing *P. australis* and *T. domingensis* root systems was influenced by both the plant fractions (PERMANOVA, *p* = 0.0001, Supplementary Table S7B) and species (PERMANOVA, *p* = 0.0033, Supplementary Table S7B) besides their interaction (PERMANOVA, *p* = 0.0015, Supplementary Table S7B). To further asses the effect of the ‘plant species’ factor, the bacterial communities’ structure was also assessed per each fraction separately (Supplementary Table S7C). Significant differences between *Phragmites* and *Typha* were detected in fractions RS2 and RS3, while the differences suggested by PCoA analysis were not supported by statistical significance for fractions RS1 and E, possibly due to the low number of biological replicates available for this study.

The taxonomic composition of the bacterial communities is reported at the Phylum level in Figure 1C. Both *Phragmites* and *Typha* root bacterial communities were dominated by *Firmicutes*, *Proteobacteria*, *Chloroflexi*, *Bacteroidota*, *Campilobacterota* and *Actinobacteriota* phyla, present at different relative abundances in the two plant species, especially in the E fraction (Supplementary Table S6B). The endophytic bacterial community of *Phragmites* plants was in fact composed mostly by *Firmicutes* and *Proteobacteria* phyla which displayed an average relative abundance of 66 and 22%, respectively. As shown by Supplementary Figure S4, the more abundant families within these phyla were *Clostridiaceae* (on average representing 37% of the total endophytic bacterial community), *Streptococcaceae* (8.5%), *Leuconostocaceae* (7%) and *Enterobacteriaceae* (8%). On the other hand, the *Typha* endophytic bacterial community mainly included *Bacteroidota* (37%), which accounted only for 3% of the ASVs colonizing the *Phragmites* endosphere, *Firmicutes* (28%) and *Proteobacteria* (21%) phyla (Supplementary Figure S4). Within these phyla the most abundant families over the total endophytic community of *Typha* plants were *Bacteroidaceae* (35%), *Lachnospiraceae* (7.5%), and *Enterobacteriaceae* (12%) (Supplementary Figure S4). Likewise, the relative abundance of *Actinobacteriota* and *Campilobacterota* phyla was one-order of magnitude higher in the E fraction of *Typha* (11 and 2%, respectively) than *Phragmites* (2 and 0.2%, respectively). The phyla relative abundance data reported in Supplementary Table S6B showed differences also in the RS1 fraction of the two plant species. Particularly, *Campilobacterota* and *Fusobacteriota* were more abundant in *Typha* (9 and 2%) than *Phragmites* (0.2 and 0.05%), while the relative abundance of *Verrucomicrobiota* was higher in *Phragmites* (5%) than *Typha* (1%). The families comprising the culturable endophytic bacteria isolated from the two plant species, namely *Enterobacteriaceae*, *Streptococcaceae*, *Leuconostocaceae* and *Weeksellaceae*, were also detected in different fractions of their root systems by 16S rRNA Illumina (Supplementary Table S6C).

**Figure 3:**
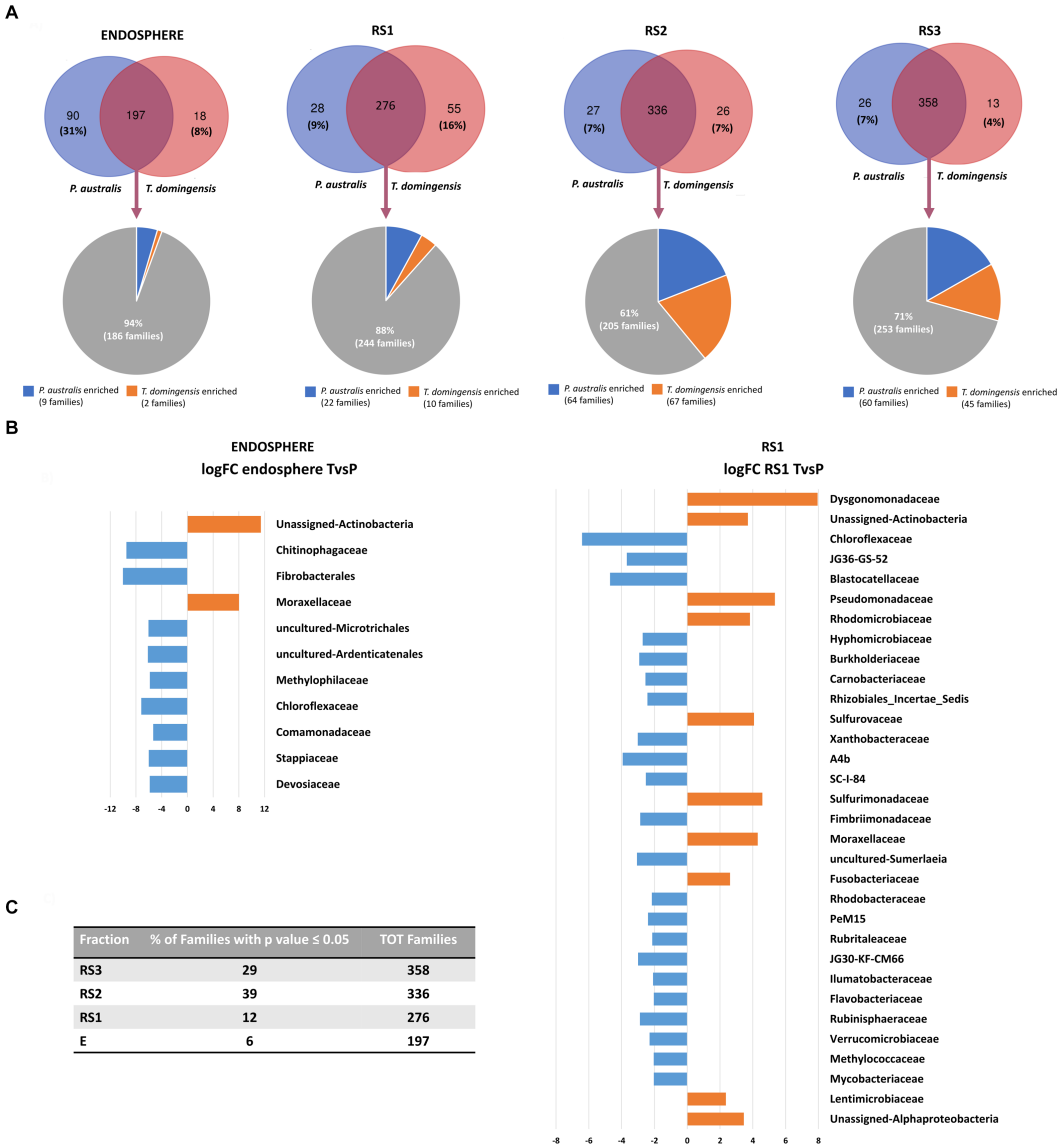
Composition of *Phragmites australis* and *Typha domingensis* bacterial communities in terms of families, according to the 16S rRNA amplicon sequencing. **(A)** Venn diagrams show numbers of exclusive and shared families between *Phragmites australis* and *Typha domingensis* per each fraction (E, RS1, RS2 and RS3). **(B)** The results of the differential abundance analysis are shown for the E and RS1 fractions. **(C)** For each root system fractions, the numbers and percentage of the plant-enriched families have been indicated.

The taxonomic differences between the bacterial communities associated to the root systems of the two plant species were visualized by Venn diagrams (Figure 3A), highlighting the number of shared and exclusive families occurring in each fraction of *P. australis* and *T. domingensis*. For each comparison, families exclusively present in one of the two plant species were detected. The selection of specific bacterial families by the plant species was evident in particular for the RS1 (16% of RS1 families exclusively associated to *T. domingensis*) and E (31% of E families exclusively associated to *P. australis*) fractions. For all fractions, the shared families were subjected to a differential abundance analysis to identify the bacterial taxa enriched by the two plant species (Supplementary Table S8). The results showed a significant variation of 29% (RS3), 39% (RS2), 12% (RS1) and 6% (E) of bacterial families between the plant species in the considered root system fractions (Figure 3C; Supplementary Tables S8A,B). A graphical focus on the bacterial families enriched in E and RS1 fractions of *P. australis* and *T. domingensis* is included in Figure 3B with the relative logFC values, while the complete list of differentially abundant families resulted from the analysis is shown in Supplementary Table 8 for all fractions, including RS2 and RS3.

Functional diversity of the bacterial communities associated to *P. australis* and *T. domingensis* root system was inferred based on the data generated by 16S rRNA gene amplicon sequencing. The analysis showed a significant variation of the number of KEGG orthologues (Supplementary Table S9A) related to several metabolic pathways (e.g., amino acid metabolism, energy metabolism, lipid metabolism, metabolism of cofactors and vitamin, metabolism of terpenoids and polyketides, Supplementary Figure S5A) according to the “plant species” factor (Supplementary Table S9B). Additional metabolic pathways (i.e., biosynthesis of other secondary metabolites, carbohydrate metabolism, nucleotide metabolism, xenobiotics biodegradation and metabolism) were differently present in the E and RS1 fractions of the two plant species according to Tukey–Kramer post-hoc test (Supplementary Tables S9B,C; Supplementary Figure S5B).

### 3.3 Antibiotic resistance genes (ARGs) spread and abundance in the root systems of *Phragmites australis* and *Typha domingensis*

**Figure 4:**
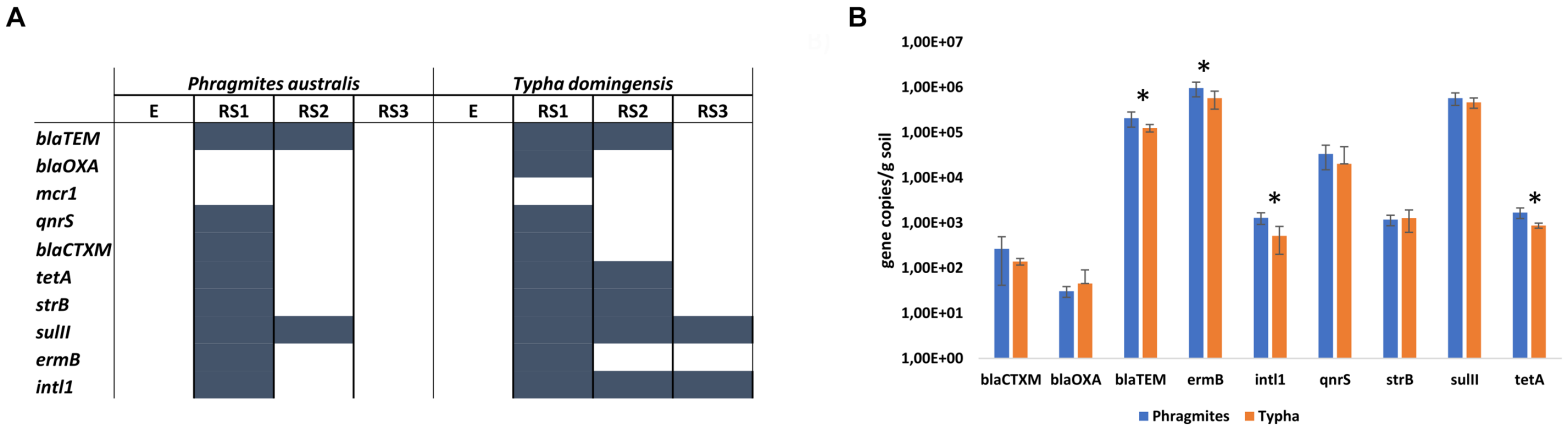
Antibiotic resistance genes in the root systems of *Phragmites australis* and *Typha domingensis*, investigated in terms of **(A)** ARGs occurrence in the four fractions and **(B)** ARGs abundance in the RS1 fraction according to qPCR results.

Qualitative PCR revealed the presence of *intl*1 gene and ARGs encoding the resistance against MLS, beta-lactams, sulfonamides, tetracyclines, aminoglycosides, colistin and quinolones in specific fractions of both *Phragmites* and *Typha* root systems (Figure 4A). No ARGs were detected in E and RS3 fractions, except for *sul*II and *intl*1 genes that were amplified in the *Typha* RS3 samples. On the contrary, all ARGs but *mcr*1 gene were detected by PCR in the RS1 fractions of at least one (*bla*_OXA_ gene) or both plant species. The *Typha* RS2 fraction was characterized by the presence of a higher number of ARGs (*bla*_TEM_, *tet*A, *str*B, *sul*II, harboring the resistance to different classes of antibiotics) and *intl*1 gene, compared to the *Phragmites* one, where only *bla*_TEM_ and *sul*II genes were detected.

The results of the qualitative assays revealed the occurrence of a higher number of ARGs in the RS1 samples. Therefore, qPCR assays were conducted on these samples to quantify the abundance of the detected genes and explore potential differences between the two plant species. The highest ARG copy numbers were found for the MLS resistance gene *erm*B, the sulfonamide resistance gene *sul*II, and the beta-lactam resistant gene *bla*_TEM_, which showed each average values of more than 10^5^ copies per gram of soil (Figure 4B). The other two beta-lactam resistant genes considered in this study, *bla*_OXA_ and *bla*_CTXM_, were detected at lower number of copies per gram of soil (10^1^ and 10^2^, respectively) for both plant species samples. Fluoroquinolone resistance gene *qnr*S and aminoglycoside resistance gene *str*B were detected at 10^4^ and 10^3^ copies per gram of soil, respectively. Significant differences (*p* value <0.05, t-student test) between RS1 sample of *Phragmites* and *Typha* plants were detected in terms of abundance for *intl*1 (1.29×10^3^ ± 3.76 ×10^2^ and 5.18×10^2^ ± 3.17 ×10^2^ gene copies/gram of soil, respectively), *tet*A (1.69×10^3^ ± 4.41 ×10^2^ and 8.74×10^2^ ± 1.15 ×10^2^ gene copies/gram of soil, respectively), *erm*B (9.6×10^5^ ± 3.46 ×10^5^ and 5.75×10^5^ ± 2.47 ×10^5^ gene copies/gram of soil, respectively) and *bla*_TEM_ (2.08×10^5^ ± 7.70 ×10^4^ and 1.26×10^5^ ± 2.36 ×10^4^ gene copies/gram of soil, respectively) genes. In addition, to estimate the size of the bacterial community hosting the detected ARGs, the 16S rRNA gene was quantified in the RS1 fractions of both *Phragmites* and *Typha* samples, measuring an average value of 6.51×10^5^ ± 2.96 ×10^5^ and 3.88×10^5^ ± 1.66 ×10^5^ gene copies/gram of soil, respectively (*p* value = 0.033, *t*-student test).

## 4 Discussion

Here, we analyzed the bacterial communities associated to the root systems of *Phragmites australis* and *Typha domingensis* plants collected from a CW system receiving primary treated wastewaters, which performances have been monitored from 2004 to 2016, demonstrating the removal of nutrients, fecal coliforms and heavy metals at level that allowed the reuse of treated water for land reclamation and crop production (Rashed, 2017). At the end of 2016, *P. australis* and *T. domingensis* root systems were collected with the primary aim to isolate bacterial strains of potential interest for microbial assisted phytodepuration. The established strain collections were characterized by a strong dominance of few bacterial genera, different according to the considered plant species, which presence could be related to the wastewaters of mixed origin streaming into this CW. The isolation of lactic acid bacteria using a nonspecific medium (98% of the isolates from *Phragmites* root endosphere) could be explained by the fact that the CW influent included wastewaters produced by small factories located in the area and producing milk and dairy products. On the other hand, the genera abundantly isolated from *Typha* root endosphere, namely *Klebsiella* and *Chryseobacterium*, likely derived from the municipal effluent present in the mixed wastewater treated in the CW.

Plant growth promoting and emerging organic pollutant degrading bacteria selected from the endosphere and the rhizosphere of constructed wetland plant species have been previously successfully applied (Syranidou et al., 2016; Salgado et al., 2018; Riva et al., 2019). However, the taxonomic identification of the isolates obtained in the present work indicated them as poorly suitable for phytodepuration application. Lactic acid bacteria are well known for the application in food industry (Wang et al., 2021) and as probiotic for human health (Quinto et al., 2014) but no information about their use in wastewater phytodepuration is reported in literature, possibly because they are uncommon in such environment. On the other hand, the use of bacterial inoculants belonging to genera that include human pathogens, such as *Klebsiella* and *Chryseobacterium,* is not recommended in compliance with the *One Health* concept (Banerjee and van der Heijden, 2023). Notably, the pathogenic potential of *Chryseobacterium* species isolated from environmental sources was recently reported (Mwanza et al., 2022) and the wide antibiotic resistance detected by MIC in all *Chryseobacterium* strains isolated in this work corroborates the risk occurring in terms of environmental safety when treated wastewater is used for irrigation purposes (Sessitsch et al., 2023).

The results of the adopted cultivation-based approach showed a sharp taxonomical difference between the endophyte collections retrieved from the two plant species, though all the sampled plants were growing in the same CW cell, received the same wastewater and thus exposed to the same seeding wastewater community, and the bacteria collections were obtained using the same medium and cultivation conditions. This marked difference was unexpected in a dynamic system such as CW environment, where root exudates are released in a substrate continuously flushed by wastewater that should mitigate the species or cultivar-specific selection of the rhizosphere microbiome, well described in soil (Reinhold-Hurek et al., 2015). This opens new perspectives considering the gap of knowledge about the selection of microbes in the root systems of plants growing in dynamic environments like constructed wetlands while the literature mostly focused on microbial communities’ composition in wastewaters (Mellado and Vera, 2021). In our study, the bacterial diversity associated to *Phragmites* and *Typha* plants was further investigated by a cultivation-independent approach considering four different fractions of the root systems, with different levels of proximity to the roots. The differences detected between the two plant species in terms of bacterial community structure highlighted the influence that plant species play during bacterial population recruitment even in the soil less intimately connected to the root (Reinhold-Hurek et al., 2015). Looking at taxa distribution, *Phragmites* endosphere was mainly composed of bacteria belonging to *Firmicutes* phylum, which comprises the families of lactic acid bacteria (i.e., *Leuconostocaceae* and *Streptococcaceae*) isolated exclusively from this plant species. *Bacteroidetes* and *Actinobacteriota* were instead detected at higher relative abundance in *Typha* endosphere. Similar differences in the bacterial communities associated to the roots of these plant species were detected by Li et al. (2013), though they used a different molecular technique, investigating geographically separated wetlands and collecting root samples during a different season compared to this study. Moreover, Li et al. (2013) detected a lower richness for *Typha* root-associated bacterial communities, in agreement to the alpha-diversity analysis conducted here on *Typha* and *Phragmites* endosphere. In each root system fraction, we detected several unique bacterial families specifically recruited by *Phragmites* or *Typha* plants and the differential abundance analysis applied on the common bacterial families revealed, in each fraction, significantly different distribution of several taxa. These bacterial families can include bacteria endowed with different functionalities of possible interest for phytodepuration process of specific wastewaters as we inferred by the Tax4Fun analysis. Additionally, the data suggest that certain macrophytes can differentially enrich in the root system bacterial species known as opportunistic pathogens. For instance, the *Moraxellaceae* family, including troublesome pathogens like *A. baumannii* (Peleg et al., 2008), was detected among the families enriched in the root tissues and rhizosphere by *Typha*, where it represents on average the 4 and 3% of the bacterial community, respectively (versus relative abundance values of 0.02 and 0.14% found in *Phragmites* endosphere and rhizosphere). Notably, additional human-related families (i.e., *Clostridiaceae* and *Bacteroidaceae*), which are rarely detected at high relative abundance in the root system microbiome, were among the main taxa associated to the two macrophytes analyzed in this study. The prevalence of these bacterial families could be explained by the origin of the wastewater treated in the CW and was different in the *Phragmites* and *Typha* endosphere. In particular, *Clostridiaceae* represented on average the 37% of the endophytic community of *Phragmites* versus the 5% in *Typha*, while *Bacteroidaceae* constituted the 35% of the endophytic community of *Typha* versus the 1% in *Phragmites*.

In terms of antibiotic resistance occurrence and spread, our investigation showed remarkable differences between the analyzed root system fractions. In both plant species, the rhizosphere was in fact the root system fraction where the highest number of ARGs, together with the *intl*1 gene, was detected, followed by the RS2 fraction. Furthermore, the ARGs and *intl*1 gene quantitative analysis conducted on the rhizosphere samples revealed significantly higher abundance of total bacteria and *erm*B, *bla*_TEM_, *tet*A and *intl*1 genes in *Phragmites* than *Typha* samples. This result can be explained by their different root morphology, which is a key factor influencing microbial density in CWs (Gagnon et al., 2007). In particular, at microcosm scale it has been proved that root surface is significantly correlated to bacterial abundance, to microbial aerobic respiration and to the activity of different enzymes acting on organic molecules (Gagnon et al., 2007). At spatial level the root distribution can be different according to the considered species, thus influencing the abundance and activity of the microbiome associated both to the plant and the substrate in CWs. Based on its higher level of microbial abundance and activity compared to the bulk soil, the rhizosphere has been indicated as a hot spot for Horizontal Gene Transfer (Sessitsch et al., 2023) and the available data cannot rule out if the higher abundance of specific ARGs in the rhizosphere of the *Phragmites* plants detected in this study are the results of higher HGT event frequencies. However, we cannot exclude that the different antibiotic resistance profiles detected in the microbiome associated to these two plant species is driven by their different composition in terms of bacterial populations. Indeed, the bacterial community structure was the primary factor driving ARG profiles and abundance in the microbiome of different vegetable grown in the same soil (Guo et al., 2021). Root exudate patterns have been suggested to play a role in this context (Chen et al., 2023), according to their recognized influence in shaping the structure of bacterial communities associated to the root system of different plant species. Furthermore, the presence of certain pollutants can determine a change in root exudation by plants (Rolli et al., 2021, 2024) influencing the recruitment of specific bacterial populations and possibly the abundance of ARGs. All in all, the collected data suggest that macrophytes growing in CWs could be able, to a different extent, to remove ARGs from wastewater and retain them in the soil attached to the roots, possibly limiting their spread into the surrounding environment.

## 5 Conclusion

Considering that plant-microbiome interactions have pivotal role in phytodepuration processes, this study highlighted the potential impact of plant species selection on the performance in CWs. Here, the microbiome associated to *Phragmites* and *Typha* root systems receiving the same wastewater showed remarkable differences in terms of phylogenetic composition and antibiotic resistance profile, an aspect that requires further investigation to clarify a possible different ability of the holobiont to remove emerging contaminant from wastewater. Indeed, measurements of antibiotic resistance determinants like ARGs by qPCR approaches would be feasible in monitoring trials and combined by the detection of pharmaceuticals in the influent and effluent of WWTPs would provide valuable information to meet the more recent EU legislation about water reuse. Hitherto, literature has overlooked the systemic evaluation of the holobiont performances in phytodepuration and we showed that further research on plant-microbiome interactions is required to allow the selection of the best performing combinations and design CW systems for the treatment of specific wastewaters.

## Data availability statement

The datasets presented in this study can be found in online repositories. The names of the repository/repositories and accession number(s) can be found in the article/Supplementary material.

## Author contributions

VR: Data curation, Formal analysis, Investigation, Visualization, Writing – original draft. LV: Data curation, Formal analysis, Writing – review & editing. AR: Resources, Writing – review & editing. AS: Resources, Writing – review & editing. RS: Resources, Writing – review & editing. AC: Resources, Writing – review & editing. EC: Resources, Writing – review & editing. FM: Conceptualization, Formal analysis, Funding acquisition, Project administration, Writing – original draft. SB: Conceptualization, Funding acquisition, Supervision, Writing – review & editing.
