# Supplementary material for: Plant species influences the composition of root system microbiome and its antibiotic resistance profile in a constructed wetland receiving primary treated wastewater
Source: Front Microbiol. 2024 Jul 24;15:1436122. doi: 10.3389/fmicb.2024.1436122 (PMC11303162; doi:10.3389/fmicb.2024.1436122)
Supplement: Supplementary file 1 [file Data_Sheet_1.docx]

Supplementary Material

**Plant species influences the composition of root system microbiome and its antibiotic resistance profile in a constructed wetland receiving primary treated wastewater.**

**Valentina Riva^1^, Lorenzo Vergani^1^, Ahmed Ali Rashed^2^, Aiman El Saadi^2^, Raffaella Sabatino^3,4^, Andrea Di Cesare^3,4^, Elena Crotti^1^, Francesca Mapelli^1^*, Sara Borin^1^**

^1^Department of Food, Environmental and Nutritional Sciences (DeFENS), University of Milan, Milan, Italy

^2^National Water Management and Irrigation Systems Research Institute, National Water Research Center, Egypt

^3^National Research Council of Italy – Water Research Institute (CNR-IRSA) Molecular Ecology Group (MEG), Verbania, Largo Tonolli 50, 28922 Verbania, Italy

^4^ National Biodiversity Future Center (NBFC), Piazza Marina 61, 90133 Palermo, Italy

*** Correspondence:**Francesca Mapelli
francesca.mapelli@unimi.it

**SUPPLEMENTARY FIGURES**

**Supplementary Figure 1. A)** Schematic layout of Lake Manzala Treatment Engineered Wetland System; **B)** Environmental parameters of the sampling site: temperature variation recorded during winter 2016 (collection time) in Port Said.


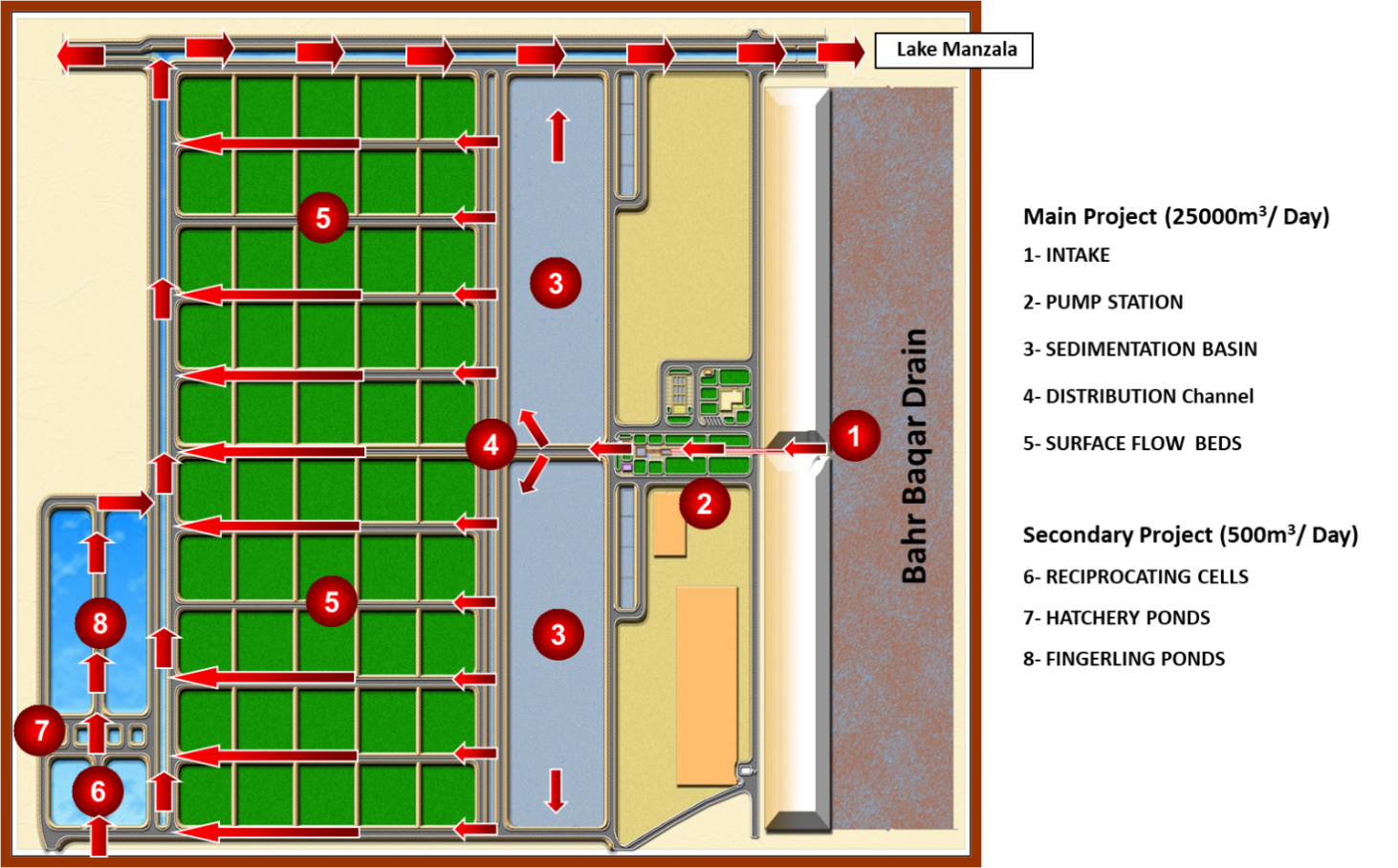


**A)**

**
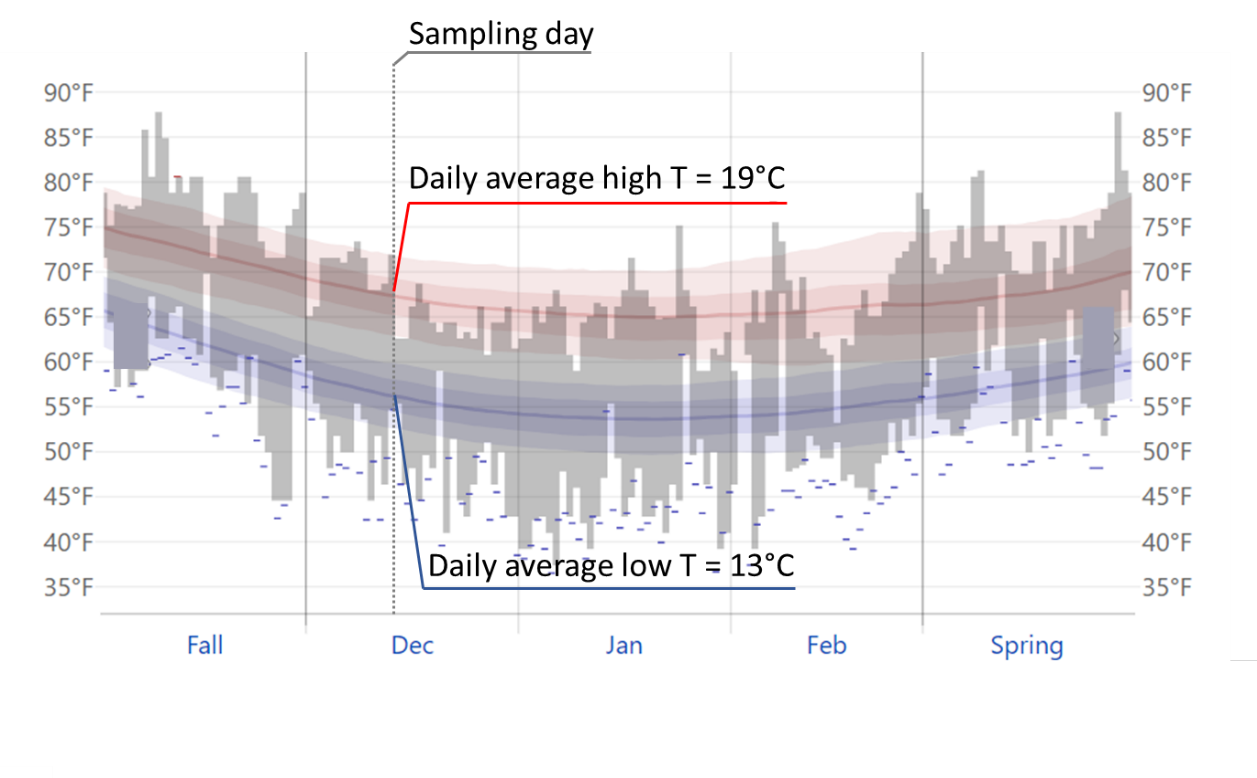
**

**B)**

**Supplementary Figure 2:** Rarefaction curve of the 16S rRNA gene Illumina libraries calculated for each sample.

**
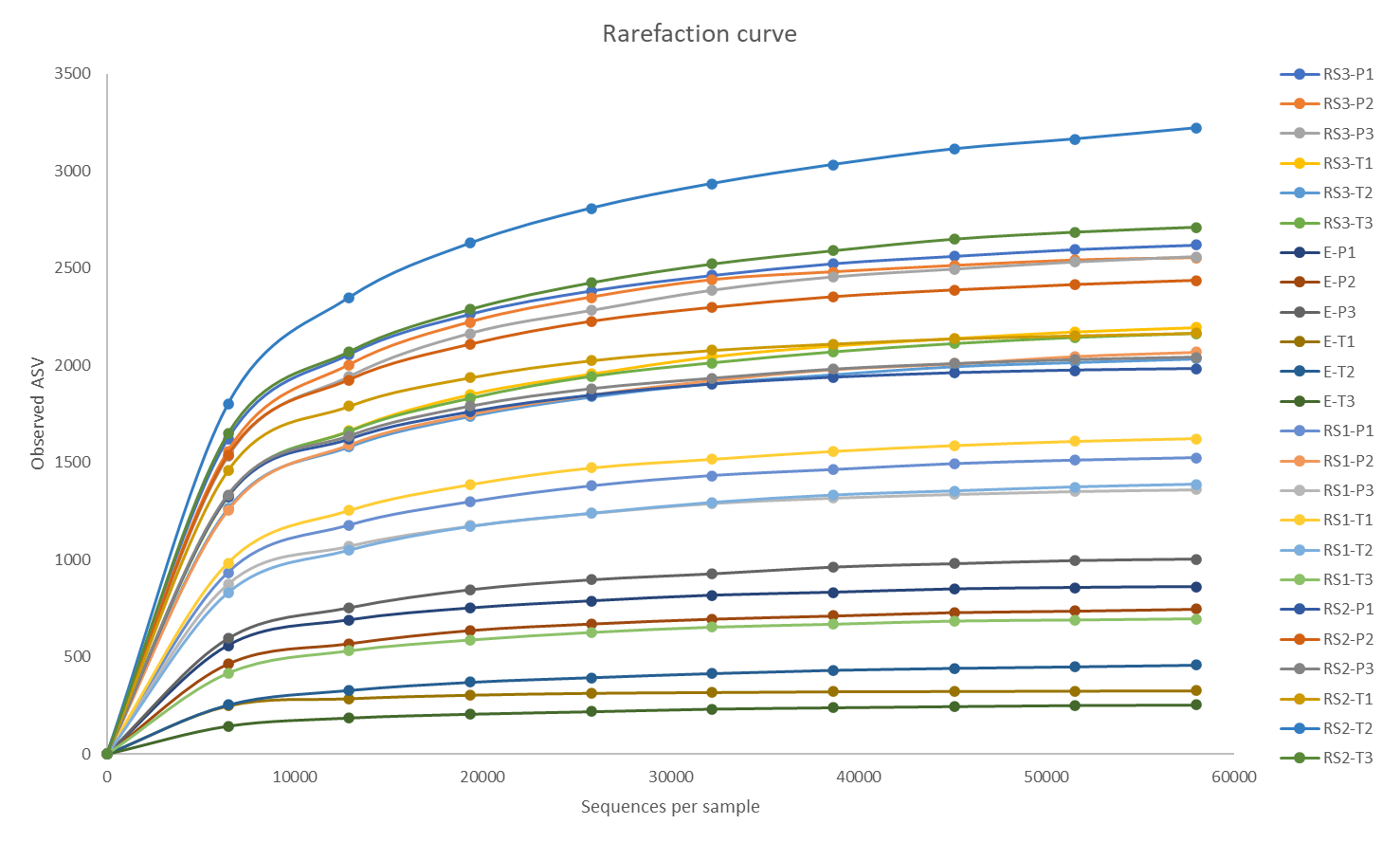
**

**Supplementary Figure 3.** α-Diversity indices of bacterial communities associated to *Phragmites australis* and *Typha domingensis*. **A)** Richness, expressed as number of ASVs, **B)** Shannon diversity, and **C)** Dominance indices. The indices were calculated from the ASV table generated by 16S rRNA gene Illumina sequencing of the bacterial community inhabiting the root endosphere (E), rhizosphere (RS1), root system 2 (RS2) and root system 3 (RS3) fractions. Letters indicate the statistical differences among the fractions of each plant species, according to ANOVA and Tukey tests. Stars indicate statistical differences between plant species, according to t-student test.


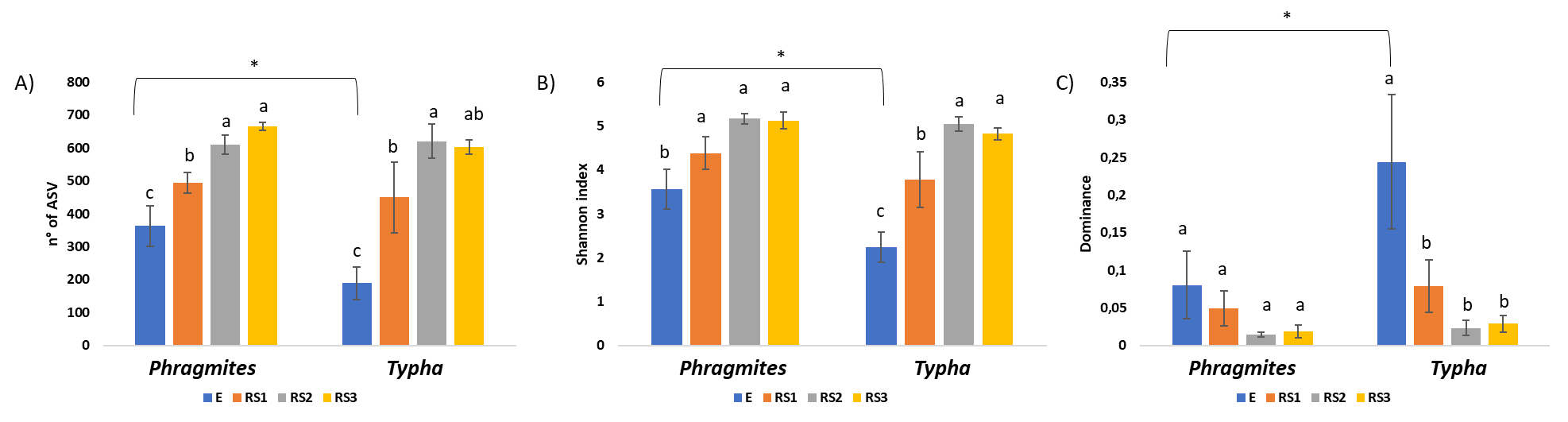


**Supplementary Figure 4.** Bacterial microbiota composition of *P. australis* (P) and *T. domingensis* (T) across fractions as detected by 16S rRNA amplicon sequencing analysis. Relative abundance of the main families is reported as mean of replicates (n = 3) for each category (fraction per plant species). Taxonomic groups with relative abundance < 1% over the total dataset are summed and reported as “other”.


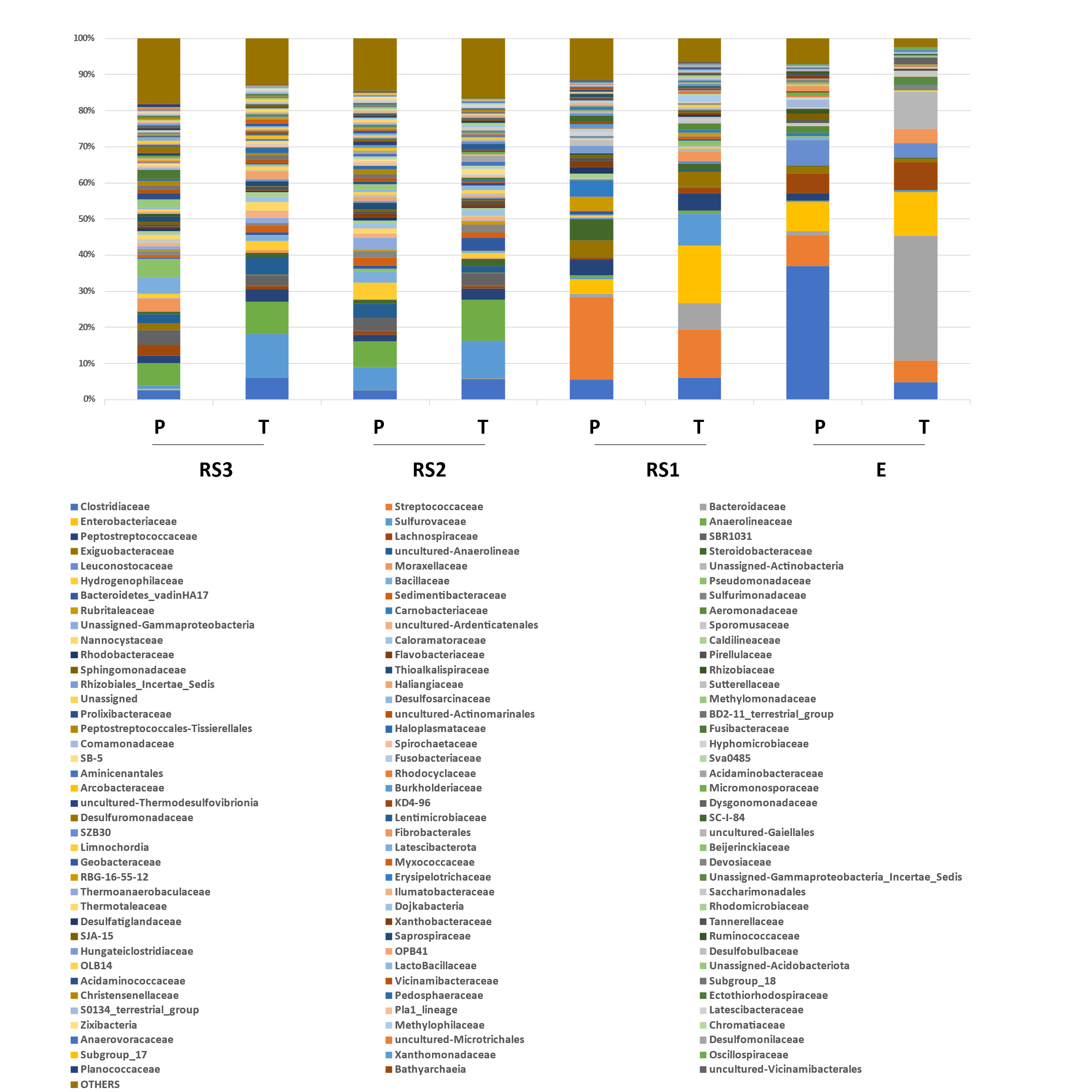


**Supplementary Figure 5.** Metabolic pathways inferred based on the data generated by 16S rRNA gene Illumina sequencing that showed a significant variation of the number of KEGG (Kyoto Encyclopedia of Genes and Genomes) orthologues among the A) plant species and B) plant species considering the different plant fractions where *p value* was <0.05 for the interaction ‘Plant species:Fraction’, according to two-ways ANOVA analysis. P= *Phragmites australis*; T= *Typha domingensis*. Statistically significant differences are indicated by stars.

**
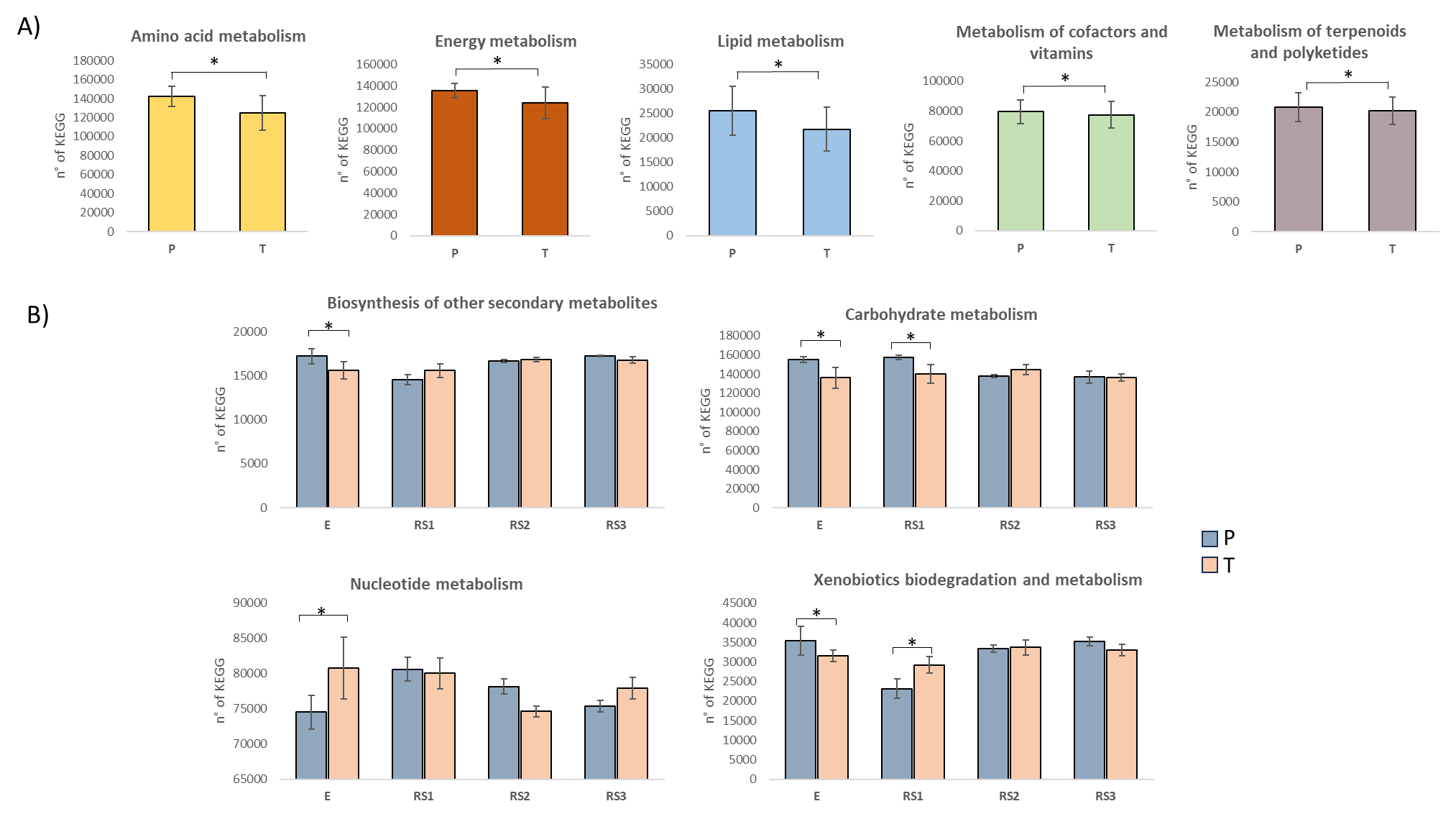
**

**SUPPLEMENTARY TABLES**

**Supplementary Table 1.** Water quality parameters at Lake Manzala Treatment Wetland cells (Monthly Average in 2016). Information about the water quality and the removal efficiency of the Constructed Wetland present in the treatment plant is colored in yellow.

| **Bahr El Baqar drain Initial Conditions** | | **Sedimentation Pond**  (Between Drain and wetland cells) | | | **Free water surface Wetland cells**  (After Sedimentation Pond) | | |
| --- | --- | --- | --- | --- | --- | --- | --- |
| Parameter | Influent Conc.  mg/L | Influent conc. mg/L | Effluent Conc. mg/L | Removal  Efficiency % | Influent conc. mg/L | Effluent Conc. mg/L | Removal Efficiency  % |
| **TSS**  **BOD**  **COD**  **Total P**  **Total N**  **Organic-N**  **NH_4_-N** | 85-155  46-88  77-116  3.2-5.2  13-32  4-15  5-17 | 81-158  42-85  80-118  3.2-5.2  13-33  4-15  5-17 | 39  26  53  1.2-4.3  8-12  2 - 4  3 - 5 | 62-82  43-75  33-60  69-82  33-60  50-87  60-97 | 30-36  19-26  54-62  1.2 – 4.2  8-12  2 - 4  3 - 5 | 4.9 - 8.8  6.9 – 9.5  26 - 31  0.42 – 2.6  2.4-5.8  1.9 - 2.8  2.0 – 3.1 | 85 – 76  61 - 64  56 - 61  43 - 64  52 - 70  30 - 53  33 - 38 |
| **F. Coliform MPN/100m** | 156000-256900 | 156000-256000 | 10500-15600 | 92-98 | 10500-15600 | 200-510 | 98-99 |
| **Pb** | 0.007-0.06 | 0.007-0.06 | 0.004-0.007 | 69-85 | 0.004-0.007 | <0.003 | --- |
| **Zn** | 0.006-0.008 | 0.006-0.008 | <0.001-0.005 | 59-95 | <0.001-0.005 | <0.001 | ---- |
| **Ni** | 0.007-0.012 | 0.007-0.012 | 0.001-0.007 | 58-81 | 0.001-0.007 | <0.003 | ---- |

**Supplementary Table 2.** Antibiotic concentration ranges used for MIC test, according to EFSA (2012) and CLSI (2014) guidelines. AMP=ampicillin; VAN=vancomycin; GEN=gentamycin; KAN=kanamycin; STR=streptomycin; ERY=erythromycin; CLI=clindamycin; TET=tetracycline; CLO=chloramphenicol; CIP=ciprofloxacin.

|  | **Concentration range (mg/L)** | | | | | | | | | |
| --- | --- | --- | --- | --- | --- | --- | --- | --- | --- | --- |
|  | **AMP** | **VAN** | **GEN** | **KAN** | **STR** | **ERY** | **CLI** | **TET** | **CLO** | **CIP** |
| ***Leuconostoc*** | 0.25-16 | - | 2-128 | 2-128 | 8-512 | 0.125-8 | 0.125-8 | 1-64 | 0.5-32 | - |
| ***Lactococcus*** | 0.25-16 | 0.5-32 | 4-256 | 8-512 | 4-256 | 0.125-8 | 0.125-8 | 0.5-32 | 1-64 | - |
| ***Enterobacter*** | - | - | 1-64 | 4-256 | 8-512 | - | - | 1-64 | 2-128 | - |
| ***Klebsiella*** | 2-128 | - | 1-64 | 4-256 | 8-512 | - | - | 1-64 | 2-128 | - |
| ***Chryseobacterium*** | 0.25-16 | 0.25-16 | 0.5-32 | - | - | - | - | - | 1-64 | 0.125-8 |

**Supplementary Table 3.** Parameters (reaction efficiency percentage and R^2^) of each qPCR assay.

| **gene** | **reaction efficiency %** | **R²** |
| --- | --- | --- |
| **blaCTXM** | 97.1 | 0.997 |
| **blaOXA** | 89.2 | 0.999 |
| **blaTEM** | 104 | 0.998 |
| **ermB** | 104.1 | 0.996 |
| **intl1** | 88.3 | 1 |
| **qnrS** | 92.3 | 0.988 |
| **strB** | 86.4 | 1 |
| **sulII** | 93.4 | 0.995 |
| **tetA** | 89.8 | 0.999 |
| **16S rRNA** | 90.4 | 0.999 |

**Supplementary Table 4.** Taxonomic identification and fingerprinting profiles (ITS and BOX) assigned to each bacterial strain isolated from A) *Phragmites australis* (n=78) and B) *Typha domingensis* (n=77) plants. The table is accessible in the Dataverse repository at the following link: https://doi.org/10.13130/RD_UNIMI/DZ4WSQ.

**Supplementary Table 5:** Results of MIC test for **A)** Lactic Acid Bacteria, **B)** *Klebsiella* spp. and **C)** *Chryseobacterium* sp. strains. MIC values (ml/L): ampicillin (AMP), vancomycin (VAN), gentamycin (GEN), kanamycin (KAN), streptomycin (STR), erythromycin (ERY), clindamycin (CLI), tetracycline (TET), chloramphenicol (CLO) and ciprofloxacin (CIP). The cut off values (mg/L) approved by EFSA 2012 are indicated. MIC values that determine resistance to a certain antibiotic are indicated in red. “nr”: not required according to EFSA (2012) and CLSI (2014) guidelines. The table is accessible in the Dataverse repository at the following link: https://doi.org/10.13130/RD_UNIMI/DZ4WSQ.

**Supplementary Table 6. A)** ASVs distribution in the analyzed samples and their taxonomical classification at the genus level. **B)** Relative abundance (%) of bacterial taxa at phylum and **C)** family level in each sample. The table is accessible in the Dataverse repository at the following link: https://doi.org/10.13130/RD_UNIMI/DZ4WSQ.

**Supplementary Table 7.** PERMANOVA analysis on beta-diversity analysis based on ASVs relative abundance in *Phragmites australis* and *Typha domingensis* root systems. A) Pair-wise PERMANOVA of ‘Fraction’ factor (4 levels: E, RS1, RS2, RS3) for each plant species. B) PERMANOVA main test of ‘Fraction’ (4 levels: E, RS1, RS2, RS3), ‘Plant spp.’ (2 levels: *P. australis, T. domingensis*) factors and their interactions on ASVs distribution. C) Pair-wise PERMANOVA of ‘Plant spp.’ factors for each fraction type. Significant differences and interactions are indicated by asterisks. Df: degrees of freedom; MS: mean sum of squares; Pseudo-F: F value by permutation; P: p statistic.

| **(A)** *P. australis* | | | | |  |  |  |  |
| --- | --- | --- | --- | --- | --- | --- | --- | --- |
| Groups | **t** | **P** | | |  |  |  |  |
| RS3, E | 3.0848 | 0.008 | | |  |  |  |  |
| RS3, RS1 | 3.945 | 0.0022 | | |  |  |  |  |
| RS3, RS2 | 1.934 | 0.0377 | | |  |  |  |  |
| E, RS1 | 2.2052 | 0.0228 | | |  |  |  |  |
| E, RS2 | 3.4168 | 0.0049 | | |  |  |  |  |
| RS1, RS2 | 4.363 | 0.0016 | | |  |  |  |  |
| *T. domingensis* | | | | | |  |  |  |
| Groups | **t** | **P** | | | |  |  |  |
| RS3, E | 2.5298 | 0.0153 | | | |  |  |  |
| RS3, RS1 | 2.4136 | 0.0185 | | | |  |  |  |
| RS3, RS2 | 2.6967 | 0.0088 | | | |  |  |  |
| E, RS1 | 1.4151 | 0.1553 | | | |  |  |  |
| E, RS2 | 2.4863 | 0.0174 | | | |  |  |  |
| RS1, RS2 | 2.0252 | 0.0486 | | | |  |  |  |
| **(B)** | | | **df** | **MS** | | | **Pseudo-F** | ***P*** |
| Fractions | | | 3 | 5797.8 | | | 10.251 | 0.0001* |
| Plant spp. | | | 1 | 2116.4 | | | 3.7418 | 0.0033* |
| Fractions × Plant spp. | | | 3 | 1408.4 | | | 2.4901 | 0.0015* |
| Res | | | 16 | 565.6 | | |  |  |
| Total | | | 23 |  | | |  |  |
| **(C)** *Fraction* | | | | |  |  |  |  |
| **E** | | | | |  |  |  |  |
| Groups | **t** | **P** | | |  |  |  |  |
| P, T | 1.476 | 0.1186 | | |  |  |  |  |
| **RS1** | | | | |  |  |  |  |
| Groups | **t** | **P** | | |  |  |  |  |
| P, T | 1.4221 | 0.1523 | | |  |  |  |  |
| **RS2** | | | | |  |  |  |  |
| Groups | **t** | **P** | | |  |  |  |  |
| P, T | 3.0296 | 0.007 | | |  |  |  |  |
| **RS3** | | | | |  |  |  |  |
| Groups | **t** | **P** | | |  |  |  |  |
| P, T | 2.1648 | 0.0204 | | |  |  |  |  |

**Supplementary Table 8.** Differential abundance analysis comparing shared families between *Typha* and *Phragmites* spp. bacterial communities in **A)** RS3, **B)** RS2, **C)** RS1 and **D)** E fractions. The table is accessible in the Dataverse repository at the following link: https://doi.org/10.13130/RD_UNIMI/DZ4WSQ.

**Supplementary Table 9. A)** Detection and distribution among the samples of KEGG orthologues related to different metabolic pathways. Columns indicate the KEGG orthology (KO) numbers and the number of related 16S rRNA Illumina reads retrieved in each sample; **B)** two-ways ANOVA test performed for all the pathways; **C)** Tukey-Kramer post hoc test on that pathways in which *p value* resulted <0.05 for the interaction ‘Plant species:Fraction’, according to two-ways ANOVA analysis. The table is accessible in the Dataverse repository at the following link: https://doi.org/10.13130/RD_UNIMI/
